# Enhanced expression of microtubule-associated protein 7 functioned as a contributor to cervical cancer cell migration and is predictive of adverse prognosis

**DOI:** 10.1186/s12935-020-01446-x

**Published:** 2020-07-29

**Authors:** Ning Tang, Dan Lyu, Jian-Fang Chang, Zhi-Tao Liu, Yan Zhang, Hai-Ping Liu

**Affiliations:** 1 Reproductive Medicine Center, The 960th Hospital of the PLA Joint Logistics Support Force, No. 25 Shifan Road, Tianqiao District, Jinan, Shandong 250031 People’s Republic of China; 2 Department of Pain Management, Tianjin First Center Hospital, Tianjin, 300192 People’s Republic of China

**Keywords:** Microtubule-associated protein 7, Prognosis, Viability, Migration, Apoptosis

## Abstract

**Background:**

Cervical cancer (CC) is one of the most common female malignancies over the world. Microtubule-associated protein 7 (MAP7) belongs to the family of microtubule-associated proteins (MAPs) which involve in microtubule dynamics and are critical in several important cellular and intracellular activities. This study aimed to investigate the expression and potential role of MAP7 in CC.

**Methods:**

The expression level of MAP7 in CC tissues and normal tissues were analyzed using the data obtained from The cancer genomes atlas (TCGA) and genotype-tissue expression (GTEx) databases. The prognostic value of MAP7 in patients with CC was analyzed by Kaplan–Meier analysis, Univariate and Multivariate analyses. Moreover, the influences of MAP7 expression alteration on the viability and motility of Caski, HeLa and C-33A cells was measured by CCK8 assay, colony formation assay, scratch assay, and transwell migration and invasion assays. Flow cytometry was conducted to determine cell apoptosis. Western blot was performed to evaluate the impact of MAP7 on the expression of apoptotic-related proteins as well as mitogen-activated protein kinase (MAPK) signaling pathway-related proteins. In vivo tumorigenicity assay was performed to explore the influence of MAP7 on tumor growth.

**Results:**

Up-regulation of MAP7 was observed in CC tissues and high MAP7 expression was positively correlated with worse prognosis. Multivariate analyses suggested that MAP7 expression can be served as an independent predictor for overall survival of patients with CC. Knockdown of MAP7 markedly suppressed Caski and HeLa cell viability, migration and invasion while notably induced cell apoptosis. Furthermore, depletion of MAP7 in Caski and HeLa cells elevated the expression levels of Active-caspase 3 and Bax, but declined the level of Bcl-2. Whilst, overexpression of MAP7 in C-33A cells presented the opposite outcomes. Additionally, knockdown of MAP7 significantly decreased the phosphorylation of mitogen-activated protein kinase kinase (MEK) and extracellular signal-regulated kinase (ERK) in Caski and HeLa cells, and overexpression of MAP7 increased their phosphorylation in C-33A cells, indicating that MAP7 may regulate the MAPK signaling pathway in CC cells. In vivo assays revealed that knockdown of MAP7 remarkably repressed the growth of CC tumors.

**Conclusion:**

The results of the present study suggest that MAP7 functions as a promoter during the occurrence and progression of CC, and that MAP7 may serve as a promising therapeutic target in CC.

## Background

Cervical cancer (CC) is one of the most common female malignancies around the world and is the fourth leading cause of female tumor-related deaths [1]. The mortality of CC has obviously decreased in most developed countries due to screening programs, however, it has not been effectively controlled in most developing countries [2–4]. Currently, the treatment options for CC patients mainly include surgical ablation, chemotherapy, and radiotherapy, which possess severe side effects and are invasive, expensive and low effective [5]. Patients with late-stage or recurrent CC are usually regarded as incurable, and there are few treatments available [6, 7]. Therefore, identifying novel effective therapeutic targets and exploring efficient treatment strategy are of great significance.

Microtubule-associated proteins (MAPs) are known to participate in microtubule dynamics and are essential in many important cellular and intracellular activities such as cell motility, division and differentiation [8]. MAP7 (also known as ensconsin or E-MAP-115), which belongs to the family of MAPs [9], has been demonstrated to present a high level in epithelial cells and a moderate expression level in neuronal cells [10]. Recently, MAP7 has been reported to show significant prognostic value in many types of diseases. High MAP7 expression has been shown to be an adverse prognostic biomarker for cytogenetically normal acute myeloid leukemia [11]. Similarly, in Stage II colon cancer patients, higher expression level of MAP7 was related to worse prognosis [12]. In lung adenocarcinoma cell line A549, MAP7 was implied as a proliferation promoter, but it showed no effect on cell cycle progression and apoptosis [13]. To date, the biological roles of MAP7 in CC as well as its underlying action mechanism in CC still remain unclear.

In the present study, the prognostic value of MAP7 expression in patients with CC was initially investigated. The impact of altered MAP7 expression on CC cell viability, motility and apoptosis were explored by depletion or overexpression of MAP7 in CC cells. The potential connection between the function of MAP7 in CC cells and the mitogen-activated protein kinase (MAPK) signaling pathway was explored as well. Our study uncovers that high MAP7 expression was correlated with poor prognosis in CC patients and it may exert a role in cell viability and migration by modulating MAPK signaling.

## Methods

### Public data collection

The data of 3 normal tissues and 275 CC tissues were downloaded from The Cancer Genomes Atlas (TCGA; https://cancergenome.nih.gov/) in TCGA-CESC dataset. The expression data of another 10 normal samples were obtained from genotype-tissue expression (GTEx) database. All the above mentioned expression data were used to analyze the expression of MAP7. TCGA-CESC dataset containing data of 306 CC cases, however, only 275 CC tissues possess expression data we needed. Hence these 275 cases were selected for relative expression analysis. Among these 275 samples, 171 samples that possess complete clinical data were selected to perform Kaplan–Meier analysis, clinical relevance analysis and Cox proportional hazards analyses. These 171 patients were divided into high/low expression groups using the median of MAP7 expression (2665.09) as a cutoff.

### Cell culture

Human CC cell lines Caski and C-33A, and endocervical epithelial cell line End1/E6E7 were bought from the American Type Culture Collection. Human CC cell line HeLa was bought from the Cell Resources Center of Shanghai Institutes for Biological Science, Chinese Academy of Sciences. Cells were cultivated in Dulbecco’s Modified Eagle Medium (Gibco; Thermo Fisher Scientific, Inc.) supplemented with fetal bovine serum (10%; HyClone; GE Healthcare Life Sciences), penicillin (100 U/ml), and streptomycin (0.1 mg/ml) (Sigma‑Aldrich; Merck KGaA, Darmstadt, Germany) at 37 °C with 5% CO_2_.

### Transfection

The MAP7 siRNA1# (si-MAP7 1#; 5′-AGCCCACATGGAGTCGCTTTACTC-3′) and siRNA2# (si-MAP7 2#; 5′-CAGGCAGCTTAGGAACTAG-3′) were designed and synthesized by GENEWIZ, Inc. (Beijing, China). A scrambled siRNA (si-con; 5′-AUCAAGGAUUCGACGGACG-3′) which did not target MAP7 cDNA was used as a negative control. Lipofectamine^®^ 2000 (Invitrogen) was used to transfect MAP7 siRNAs (50 nM) and scrambled siRNA into Caski and HeLa cells following the manufacturer’s procedure. pcDNA3.1-MAP7 was constructed for overexpression of MAP7. pcDNA3.1-MAP7 (MAP7-OE) and empty pcDNA3.1 plasmid (vector) were transfected into C-33A cells using Lipofectamine^®^ 2000. After 24 h transfection, the following experiments were performed.

### Quantitative real-time polymerase chain reaction analysis (qRT-PCR)

Total RNA of End1/E6E7, Caski, C-33A and HeLa cells were extracted using an Ultrapure RNA kit (CwBio, Beijing, China). Total RNA of Caski and HeLa cells transfected with MAP7 siRNA or scrambled RNA, and C-33A cells transfected with pcDNA3.1-MAP7 or pcDNA3.1 were extracted after 24 h transfection. Then the extracted RNA were reverse transcribed to cDNA by HiFiScript cDNA Synthesis Kit (CwBio, Beijing, China). qPCR was performed on an Applied Biosystems 7300 Sequence Detection System. GAPDH expression was utilized for normalizing the results. The primers we used were: MAP7 F: 5′-CTGCTAGGTGAGGGGAACTG-3′, MAP7 R: 5′-TGCTAGTTCCTAAGCTGCCTG-3′, GAPDH F: 5′-GGAGCGAGATCCCTCCAAAAT-3′, GAPDH R: 5′-GGCTGTTGTCATACTTCTCATGG-3′. The relative expression of MAP7 was calculated using 2^−∆∆Ct^ method.

### Cell counting kit-8 (CCK8) and Colony-formation assays

Cell viability was explored using a cell counting kit-8 (CCK8, DOJINDO, Japan) at indicated time after transfection following the manufacturer’s description. The optical density (OD) was detected at the wavelength of 450 nm. The viability curves were drafted according to the OD value using a Graph Pad Prism 5 software.

For colony-formation assay, 400 cells were seeded into a 60 mm dish containing 5 ml pre-incubated medium. After culturing for 1–2 weeks, cells were fixed with 4% paraformaldehyde for 30 min and stained by 0.1% crystal violet dye for 20 min. The colonies were analyzed and counted.

### Scratch assay

At 24 h post-transfection, HeLa and Caski cells transfected with si-MAP7 1#, si-MAP7 2# or si-con and C-33A cells transfected with pcDNA3.1-MAP7 or pcDNA3.1 were added into 6-well plates (5 × 10^5^/well) and cultivated at 37 °C for 24 h to form confluent monolayer. Next, wounds were made using a 200 ml micropipette tip. The scratched cell monolayer was washed with PBS for 3 times. The plates were added with serum-free medium and kept at 37 °C with 5% CO_2_. After 0 and 24 h cultivation, images of the wounds were observed under a light microscope (magnification 100×). The widths of the wounds were measured to quantify cell migration.

### Transwell assays

We performed Transwell assays to investigate the effect of MAP7 on cervical cell mobility using the methods described previously [14, 15]. Transwell chambers coated with or without matrigel (BD Biosciences, USA) were used accordingly to perform invasion or migration assays. Cell suspensions were prepared using serum-free medium after 24 h transfection and the upper chamber was added with 10,000 or 5000 cells. The lower room was supplemented with 500 μl complete medium. After 24 h incubation, the cells in the upper chamber were removed and the invaded or migrated cells were fixed by paraformaldehyde and stained with crystal violet. Subsequently, the invaded and migrated cells were imaged using a light microscope and counted in 5 random fields (magnification 200×).

### Apoptosis assay

Cells were collected and washed by PBS, and then suspended in binding buffer to a density of 1–5 × 10^6^ cells/ml. Cell suspension (100 μl) and 5 µl of annexin V/FITC (BD Biosciences) were incubated together for 5 min in the dark. Hereafter, the tube was added with 10 µl of propidium iodide stain and 400 µl of PBS. Apoptosis was assessed using a flow cytometer (BD Biosciences, USA) and analyzed with FlowJo software 7.2 (FlowJo LLC).

### Western blot analysis

Total proteins were obtained from CC cells 48 h post-transfection using RIPA lysates (Thermo Fisher Scientific, Inc.) containing protease inhibitor. After quantifying the protein using a BCA assay (Bio-Rad Laboratories, Hercules, CA), 20 μg protein was heated and separated by 10% SDS-PAGE. Then the protein on the gel was transferred to the PVDF membranes (Millipore, Bedford, MA) and blocked by 5% skim milk for 1 h followed by being incubated with the rabbit anti-human primary anti-bodies overnight at 4 °C. The primary anti-bodies against MAP7 (Abcam; cat. no. ab251799; 1:1000), MEK (CST; cat. no. 9126; 1:5000), ERK (CST; cat. no. 9102; 1:5000), p-MEK (CST; cat. no. 2338; 1:5000), p-ERK (CST; cat. no. 4370; 1:5000), Active-Caspase 3 (CST; cat. no. 9664; 1:1000), Bax (CST; cat. no. 5023; 1:1000), Bcl-2(CST; cat. no. 4223; 1:1000) and GAPDH (CST; cat. no. 5174; 1:5000) were used. Then the goat anti-rabbit secondary anti-body (CST; cat. no. 7074; 1:2000) was used for incubation of the membranes at room temperature. The protein signals were detected using the Enhanced chemi-luminescence (ECL) plus detection kit (Thermo Fisher Scientifc, Inc.). GAPDH was utilized as internal control. Densitometry analysis was performed using Quantity One 4.6.2 software.

### In vivo tumorigenicity study

Caski cells with stable depleted MAP7 or transfected with negative control (1 × 10^7^ cells) were injected into 5-week-old, female athymic BALB/c nu/nu mice (17–19 g) which were obtained from Vital River Laboratories (Beijing, China). Tumor volume was measured and calculated at 3, 7, 14, 21 days after injection (Volume = 1/2(length × width^2^). After 21 days, the mice were euthanized to remove the tumor. The weight of the tumor was measured immediately. This study was approved by the Ethics Committee of The 960th Hospital of the PLA Joint Logistics Support Force.

### Statistical analyses

Kaplan–Meier analysis and log-rank test were performed to evaluate the relationship between MAP7 expression and overall survival of patients with CC. The samples were divided into high and low MAP7 expression groups according to the median of MAP7 expression, and Chi square (χ^2^) test was used for analysis of association between MAP7 expression and clinical features. The independent prognostic factors in CC patients were analyzed by Univariate and Multi-variate Cox proportional hazards analyses. The difference between two groups was calculated by student’s t test. One‑way ANOVA followed by Dunnett’s post hoc test was used for comparing the differences among ≥ 3 groups. All the assays were repeated for three independent times and all the data were presented as mean ± SD. P < 0.05 was considered to indicate a statistically significant difference.

## Results

### Up-regulation of MAP7 is associated with unfavorable prognosis in patients with CC

**Fig. 1:**
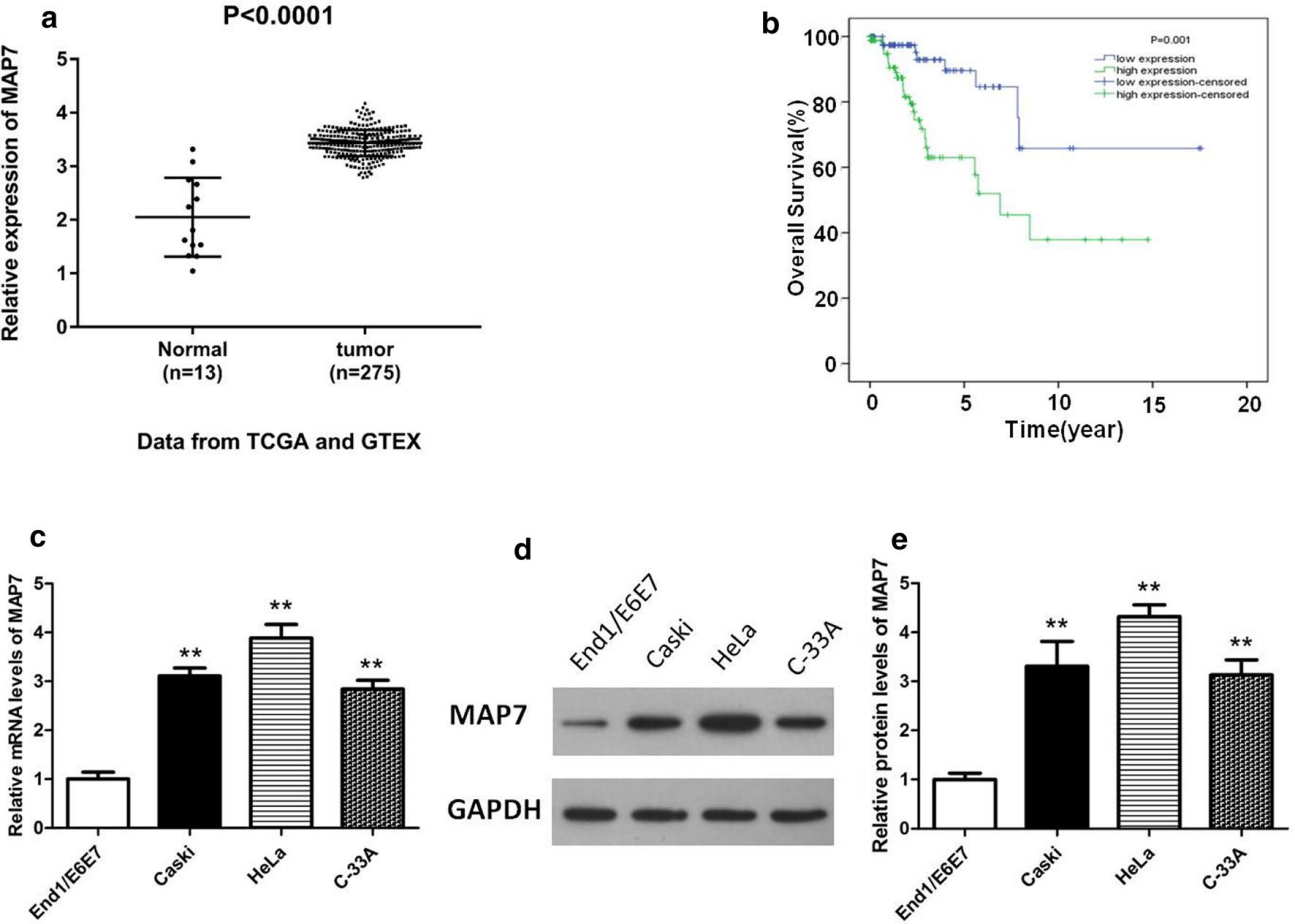
Expression of MAP7 is upregulated in CC tissues and cells, and is associated with worse prognosis of patients. **a** Analysis of MAP7 expression in patients with CC (n = 275) and healthy people (n = 13) based on data from The Cancer Genome Atlas and GTEx database. **b** Kaplan–Meier method was utilized for analysis of the correlation between MAP7 expression and overall survival time. The cutoff point for high (n = 85) and low MAP7 expression (n = 86) was the median. **c**–**e** Expression of MAP7 in cervical epithelial cells, End1/E6E7 and CC cell lines, Caski, HeLa and C-33A. **p < 0.01 vs. End1/E6E7. MAP7, microtubule-associated protein 7

The mRNA level of MAP7 in patients with CC was analyzed based on the data obtained from TCGA database and GTEx database containing 13 normal tissue samples and 275 CC tissue samples. The results indicated that MAP7 expression is remarkably up-regulated in CC tissues (Fig. 1a, p < 0.001). Among these 275 samples, 171 samples which possess complete clinical data were used to perform the following analyses. We next performed a Chi square test to evaluate the correlation between MAP7 expression and clinical-pathological factors in the 171 CC patients. As shown in Table 1, the expression level of MAP7 was significantly associated with death (p = 0.003). However, no significant relationship between MAP7 and other pathological factors was observed.

**Table 1:** Correlation between MAP7 expression and clinic-pathological features of 171 CC patients

Characteristics	Expression of MAP7		p value
	Low	High	
Age			0.508
< 60	74	70	
≥ 60	12	15	
Grade			0.494
G1 + G2	49	44	
G3 + G4	37	41	
Stage			0.180
I + II	72	77	
III + IV	14	8	
Tumor size			0.094
T1 + T2	85	80	
T3 + T4	1	5	
Lymph node metastasis			0.151
N0	55	63	
N1	31	22	
Distant metastasis			0.082
M0	83	85	
M1	3	0	
Death			0.003*
No	78	62	
Yes	8	23	

These analyses were conducted based on the data downloaded from TCGA database

* p < 0.05

The association between MAP7 expression and overall survival was then investigated in 171 patients with CC using Kaplan–Meier analysis. High MAP7 expression was significantly related to poor prognosis (p = 0.001, Fig. 1b). In addition, univariate analysis revealed that MAP7 expression (p = 0.002), tumor size (p = 0.009), and lymph node metastasis (p = 0.002) were remarkably associated with overall survival of CC patients (Table 2). These 3 factors which were significant in univariate analysis were then included in the multivariate analysis. Multivariate analysis indicated that MAP7 expression (p = 0.001) and lymph node metastasis (p = 0.002) may serve as independent predictors of worse survival of patients with CC.

**Table 2:** Univariate and multivariate analysis of prognostic factors of CC

Variables	Univariate analysis			Multivariate analysis		
	p value	HR	95% CI	p value	HR	95% CI
MAP7 expression (high/low)	0.002*	3.619	1.613–8.119	0.001*	3.824	1.691–8.650
Stage (I + II/III + IV)	0.817	0.868	0.263–2.864			
Tumor size (T1 + T2/T3 + T4)	0.009*	4.109	1.421–11.885	0.057	2.911	0.967–8.767
Distant metastasis (M0/M1)	0.653	0.048	0.000–26,846.367			
Lymph node metastasis (N0/N1)	0.002*	3146	1.539–6.435	0.002*	3.168	1.522–6.591
Age (< 60/≥ 60)	0.242	1.654	0.712–3.845			
Grade (G1 + G2/G3 + G4)	0.994	0.997	0.487–2.041			

These analyses were conducted based on the data downloaded from TCGA database

*HR* hazard ratio

* p < 0.05

### MAP7 expression is up-regulated in CC cell lines

We further analyzed the expression level of MAP7 in endocervical epithelial cell line End1/E6E7 and human CC cell lines Caski, HeLa and C-33A by qRT-PCR and Western blot. The results showed that both the mRNA and protein expression levels of MAP7 were significantly up-regulated in all tested CC cell lines compared with that in End1/E6E7 cells and HeLa showed the highest MAP7 expression level (Fig. 1c–e, p < 0.001). As C-33A presented the lowest MAP7 expression level among all the tested CC cell lines, it was selected to conduct the overexpression assays. Meanwhile, Caski and HeLa cell lines, which showed a relative higher MAP7 expression level than C-33A cells, were used to carry out the silencing assays in our following experiments.

### MAP7 exhibits a promoting role in CC cell viability

**Fig. 2:**
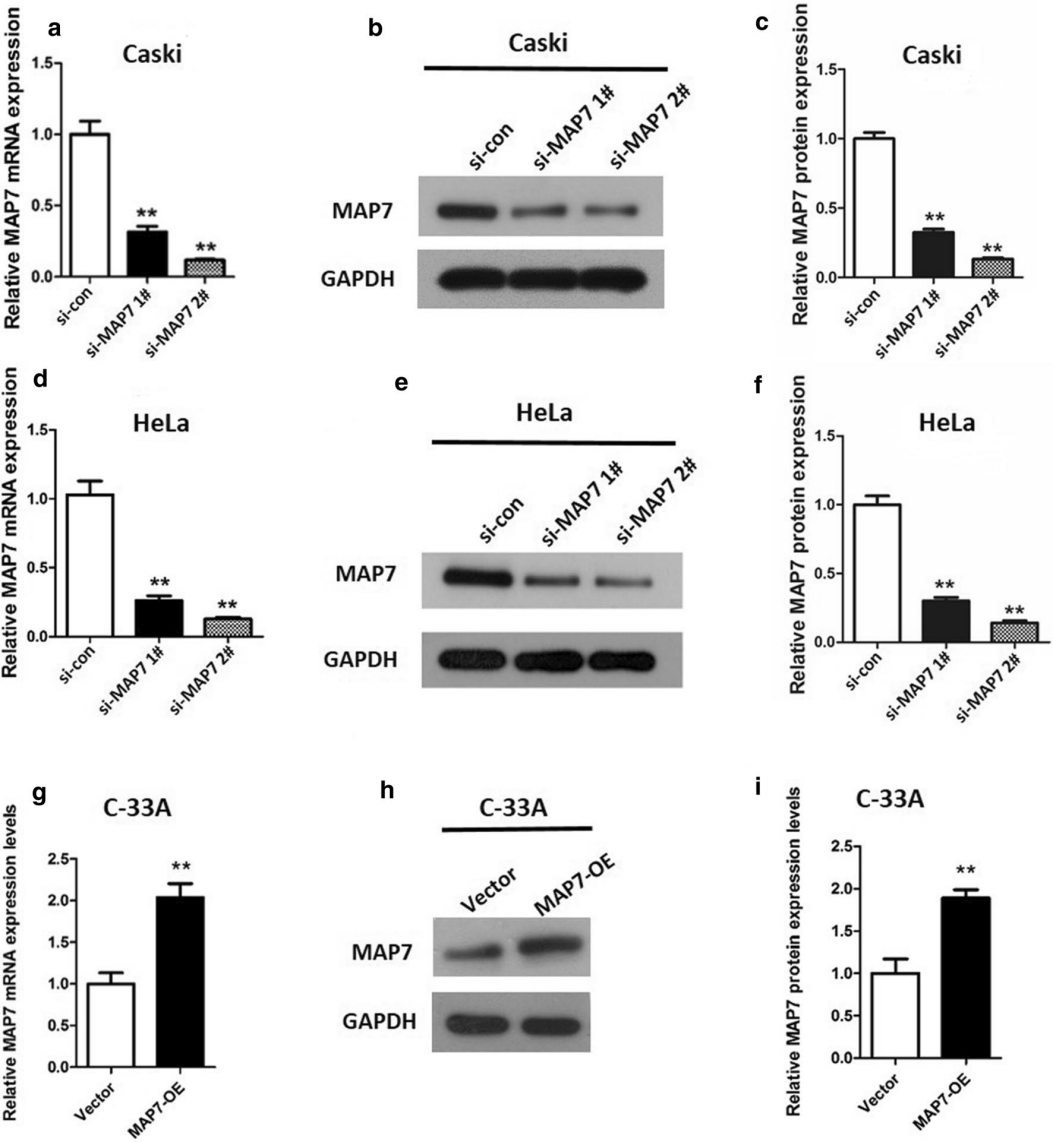
MAP7 expression in CC cells transfected with si-MAP7 1#/2# or MAP7-OE. **a** mRNA and **b**, **c** protein expression of MAP7 in Caski cells; **d** mRNA and **e**, **f** protein expression of MAP7 in HeLa cells 24 h after transfection with si-MAP7 1#/2#; and **g** mRNA and **h**, **i** protein expression of MAP7 in C-33A cells 24 h after transfection with MAP7-OE. n = 6; **p < 0.01 vs. controls (si-con or vector). MAP7, microtubule-associated protein 7; si-MAP7, siRNA targeting MAP7; si-con, scrambled siRNA; MAP7-OE, MAP7-overexpression vector

In order to study the effect of MAP7 on CC cell biological properties, MAP7 was knocked down in Caski and HeLa cells using MAP7 siRNA1# and 2#, and overexpressed in C-33A cells using pcDNA3.1-MAP7. It was obviously observed that the expression of MAP7 was markedly decreased both at RNA level (Fig. 2a, d, p < 0.01) and protein level (Fig. 2b, c, e, f, p < 0.01) in Caski and HeLa cells after transfected with MAP7 siRNAs. si-MAP7 2# showed a relative higher knockdown efficiency. On the contrary, the mRNA and protein expression levels of MAP7 were significantly up-regulated in C-33A cells after transfected with pcDNA3.1-MAP7 (Fig. 2g–i, p < 0.01).

**Fig. 3:**
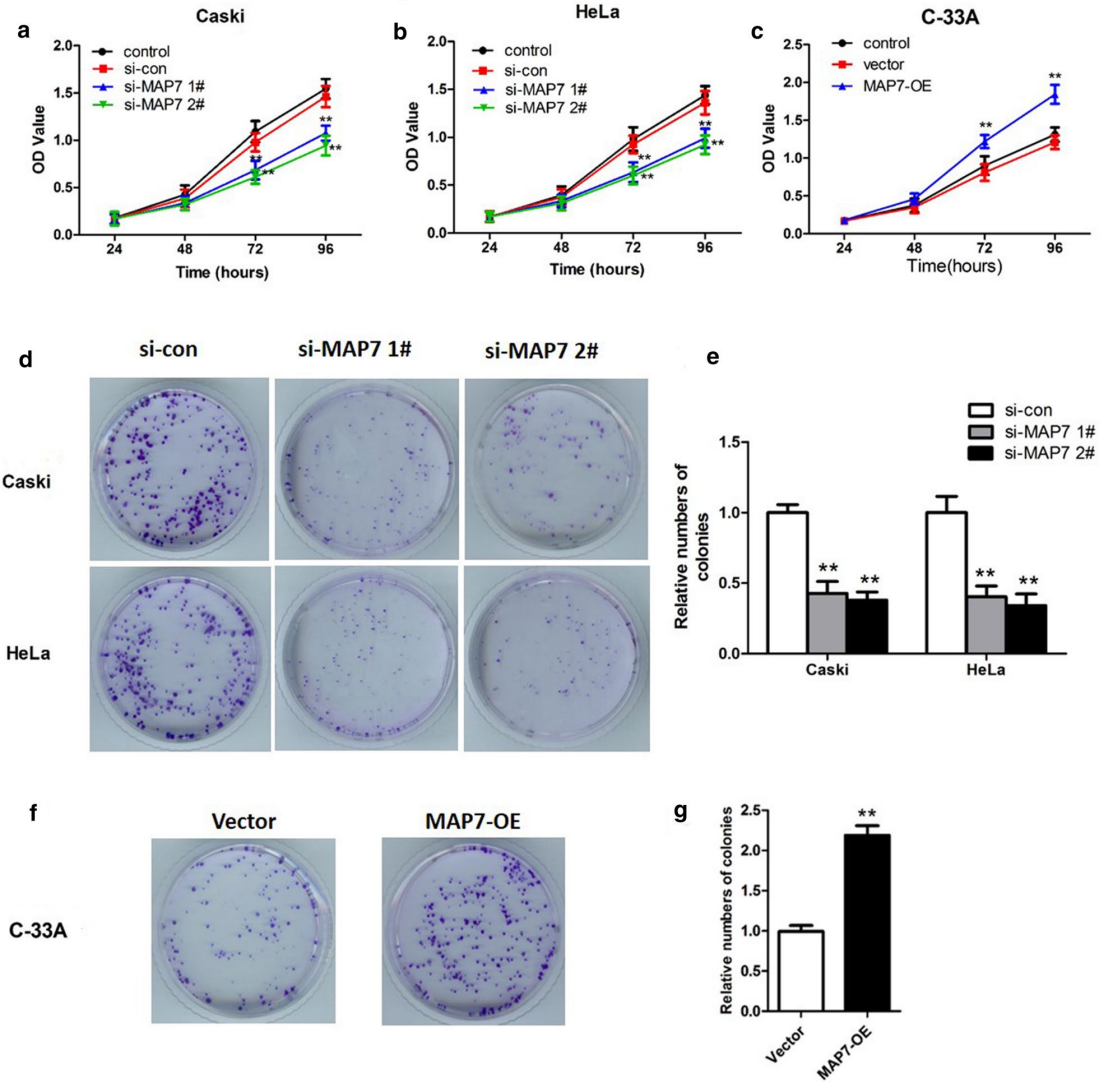
Cell viability and colony formation in CC cells transfected with si-MAP7 1#/2# or MAP7-OE. Cell viability was determined by Cell Counting Kit-8 assay in **a** Caski and **b** HeLa cells with MAP7 knockdown; and **c** C-33A cells with MAP7 overexpression. **d**, **e** Colony formation assays in Caski and Hela cells with MAP7 knockdown. **f**, **g** Colony formation assay in C-33A cells with MAP7 overexpression. n = 6; **p < 0.01 vs. controls (si-con or vector). *MAP7* microtubule-associated protein 7, *si-MAP7* siRNA targeting MAP7, *si-con* scrambled siRNA, *MAP7-OE* MAP7-overexpression vector, *OD* optical density

After transfection with si-MAP7 1# or 2# for 24 h, the viability of Caski and HeLa cells was tested using CCK8 assay and colony formation assay. The results of CCK8 assay showed that silencing MAP7 remarkably inhibited the viability of Caski (Fig. 3a) and HeLa cells (Fig. 3b) compared with cells in control group and si-con group at 72 h and 96 h (p < 0.01). As the viability of cell in control group and si-con group is similar, control group is not included in the next experiments. In colony formation assays, Caski and HeLa cells transfected with si-MAP7 1# and 2# formed fewer colonies compared with the corresponding si-con groups (Fig. 3d, e, p < 0.01). In C-33A cells with overexpression of MAP7, cell viability and colony formation were increased compared with cells transfected with empty vector (Fig. 3c, f, g, p < 0.01). These data suggested that MAP7 influenced CC cell viability.

### MAP7 exhibits a facilitating role in CC cell invasion and migration

**Fig. 4:**
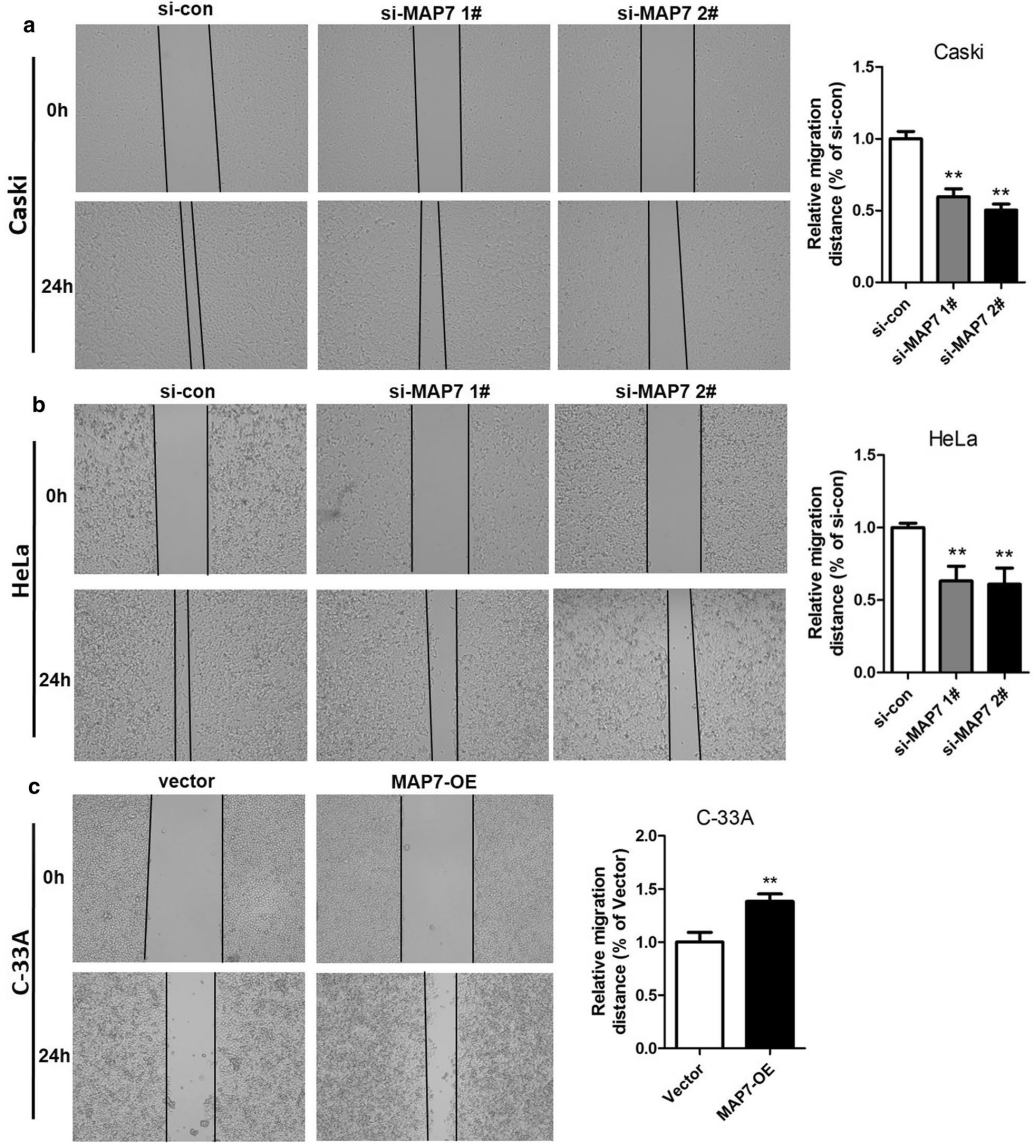
Scratch assay was conducted to detect the migratory ability of Caski and HeLa cells transfected with si-MAP7 1# or 2# and C-33A cells transfected with MAP7-OE. Representative images of scratch assays of Caski cells (**a**) and HeLa cells (**b**) with depleted MAP7 and quantification of the relative migration distance. **c** Representative images of scratch assays of C-33A cells with overexpressed MAP7 and quantification of the relative migration distance. **p < 0.01 vs. si-con groups or vector group. *MAP7* microtubule-associated protein 7, *si-MAP7* siRNA targeting MAP7, *si-con* scrambled siRNA

**Fig. 5:**
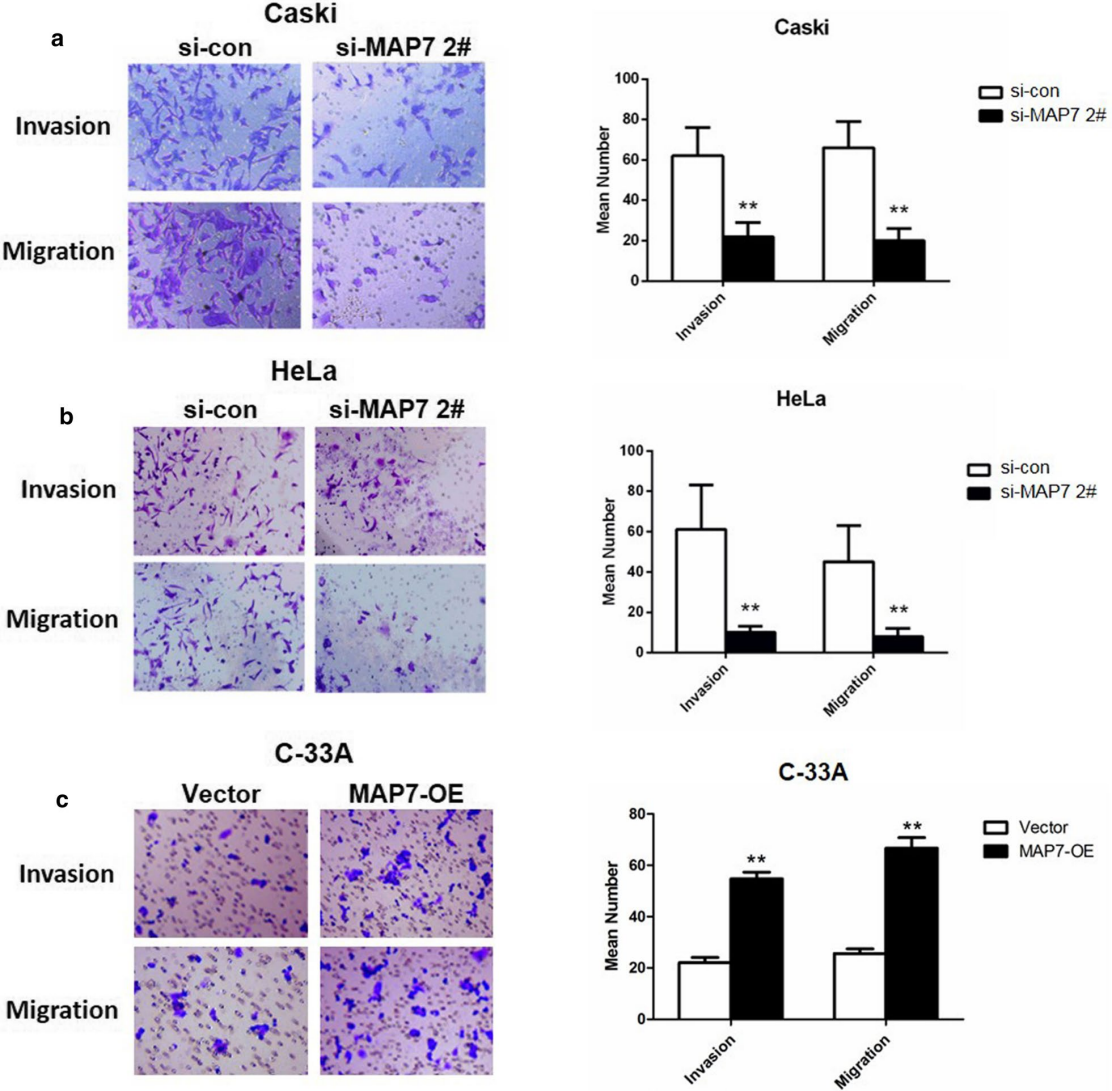
Migration and invasion in Caski and HeLa cells transfected with si-MAP7 2# and C-33A cells transfected with MAP7-OE. **a**, **b** Representative images of Transwell assays of Caski and HeLa cells with MAP7 knockdown and quantification of invading and migrating cells. **c** Representative images of Transwell assays of C-33A cells with MAP7 overexpression and quantification of invaded and migrated cells. n = 6; **p < 0.01 vs. si-con groups or vector group. *MAP7* microtubule-associated protein 7, *si-MAP7* siRNA targeting MAP7, *si-con* scrambled siRNA

We performed scratch assay to study the effect of MAP7 on CC cell motility. The results revealed that knockdown of MAP7 significantly reduced the migratory distance of Caski and HeLa cells, whilst overexpression of MAP7 dramatically enlarged the migratory distance of C-33A cells (Fig. 4a–c). Since no significant difference was found between the effect of si-MAP7 1# and si-MAP7 2# in the above experiments, si-MAP7 1# was not included in transwell assays. Consistent with the scratch assay, transwell migration assays revealed that knockdown of MAP7 significantly reduced the numbers of migrated Caski and HeLa cells (Fig. 5a, b, p < 0.01), while overexpression of MAP7 notably increased the number of migrated C-33A cells (Fig. 5c, p < 0.01). These data indicated that knockdown of MAP7 suppressed the migration of CC cells.

We then studied the effect of MAP7 on CC cell invasion by transwell invasion assay. There were fewer invading cells in Caski si-MAP7 2# and HeLa si-MAP7 2# groups compared to the si-con groups, indicating that knockdown of MAP7 inhibited CC cell invasion (Fig. 5a, b, p < 0.01). Overexpression of MAP7 in C-33A cells increased the numbers of invading and migrating cells (Fig. 5c, p < 0.01). These results suggest that MAP7 may function as a promoter of CC cell invasion and migration.

### Overexpression of MAP7 represses cell apoptosis

**Fig. 6:**
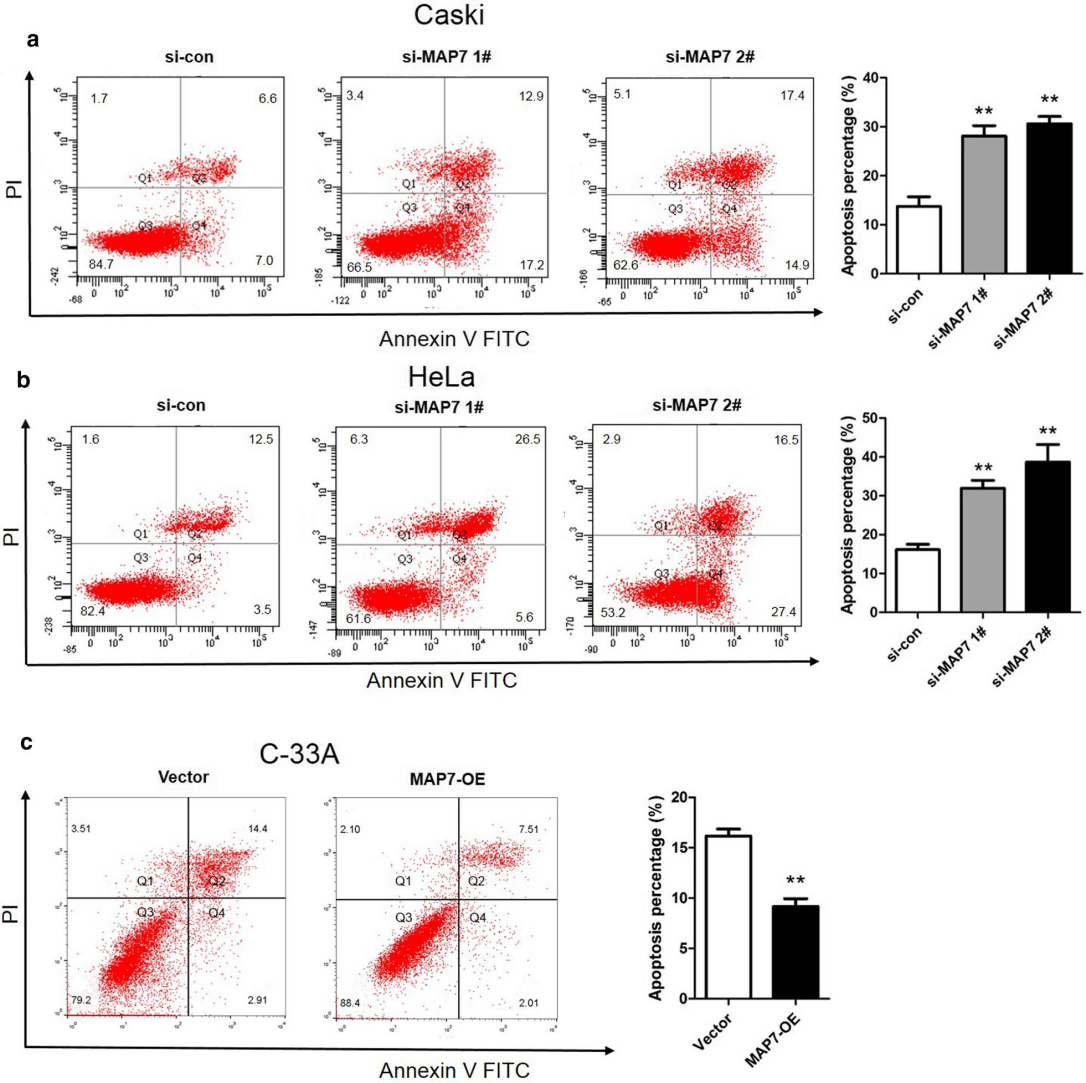
Apoptosis in CC cells transfected with si-MAP7 1#/2# or MAP7-OE. **a** Apoptosis assessed by flow cytometry in Caski cells with MAP7 knockdown and quantification of the percentage of apoptotic Caski cells. **b** Apoptosis assessed by flow cytometry in HeLa cells with MAP7 knockdown and the percentage of apoptotic HeLa cells. **c** Apoptosis in C-33A cells with MAP7 overexpression and the percentage of apoptotic C-33A cells. n = 6; **p < 0.01 vs. controls (si-con or vector). *MAP7* microtubule-associated protein 7, *si-MAP7* siRNA targeting MAP7, *si-con* scrambled siRNA, *MAP7-OE* MAP7-overexpression vector, *PI* propidium iodide

The effect of MAP7 on cell apoptosis was analyzed by Flow cytometry. The results showed that knockdown of MAP7 in Caski cells significantly increased the apoptosis percentage compared with the si-con group (p < 0.01, Fig. 6a). Similar increasing trend was observed in HeLa cells with depleted MAP7 (p < 0.01, Fig. 6b). On the contrast, overexpression of MAP7 in C-33A cells obviously reduced the apoptosis percentage compared with the vector group (p < 0.01, Fig. 6c). These phenomena hinted that MAP7 took a repressive role in CC cell apoptosis.

**Fig. 7:**
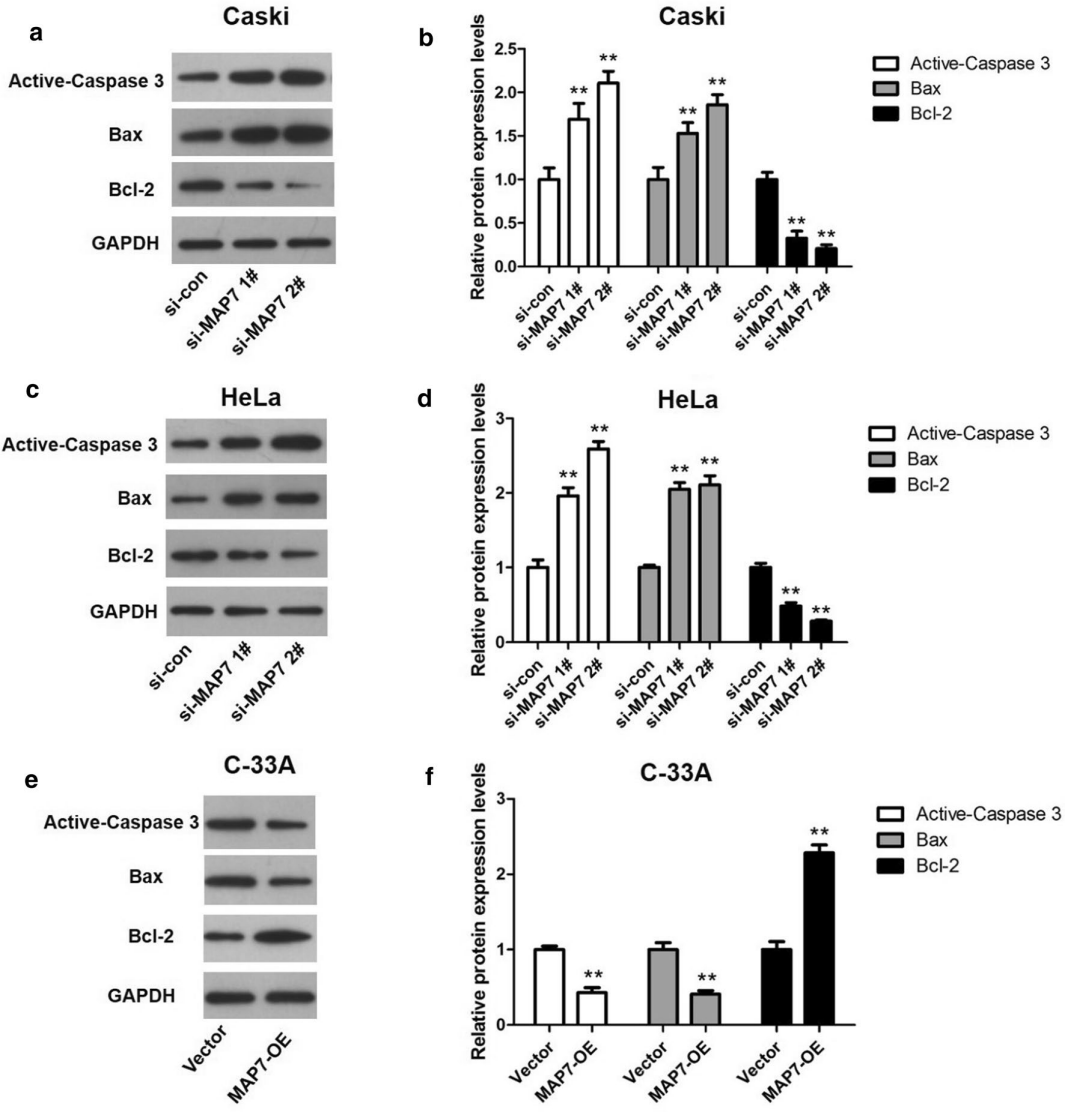
Apoptosis-related proteins were detected by Western blot. **a**, **b** The effect of MAP7 depletion on the expression of apoptosis-related proteins in Caski cells. **c**, **d** The effect of MAP7 depletion on apoptosis-related proteins in HeLa cells. **e**, **f** The effect of MAP7 overexpression on apoptosis-related proteins in C-33A cells. n = 6; **p < 0.01 vs. controls (si-con or vector)

We then detected the expression changes of the apoptosis-related proteins. As presented in Fig. 7, the expression levels of Active-caspase 3 and Bax in Caski and HeLa cells were remarkably increased while the level of Bcl-2 was significantly decreased in the si-MAP7 1# and si-MAP7 2# group compared with that in the si-con group. Furthermore, the levels of Active-caspase 3 and Bax were declined whilst the level of Bcl-2 was up-regulated in C-33A cells in the MAP7-OE group compared with that in the vector group.

### MAP7 expression changes affect the activation of MAPK signaling pathway

**Fig. 8:**
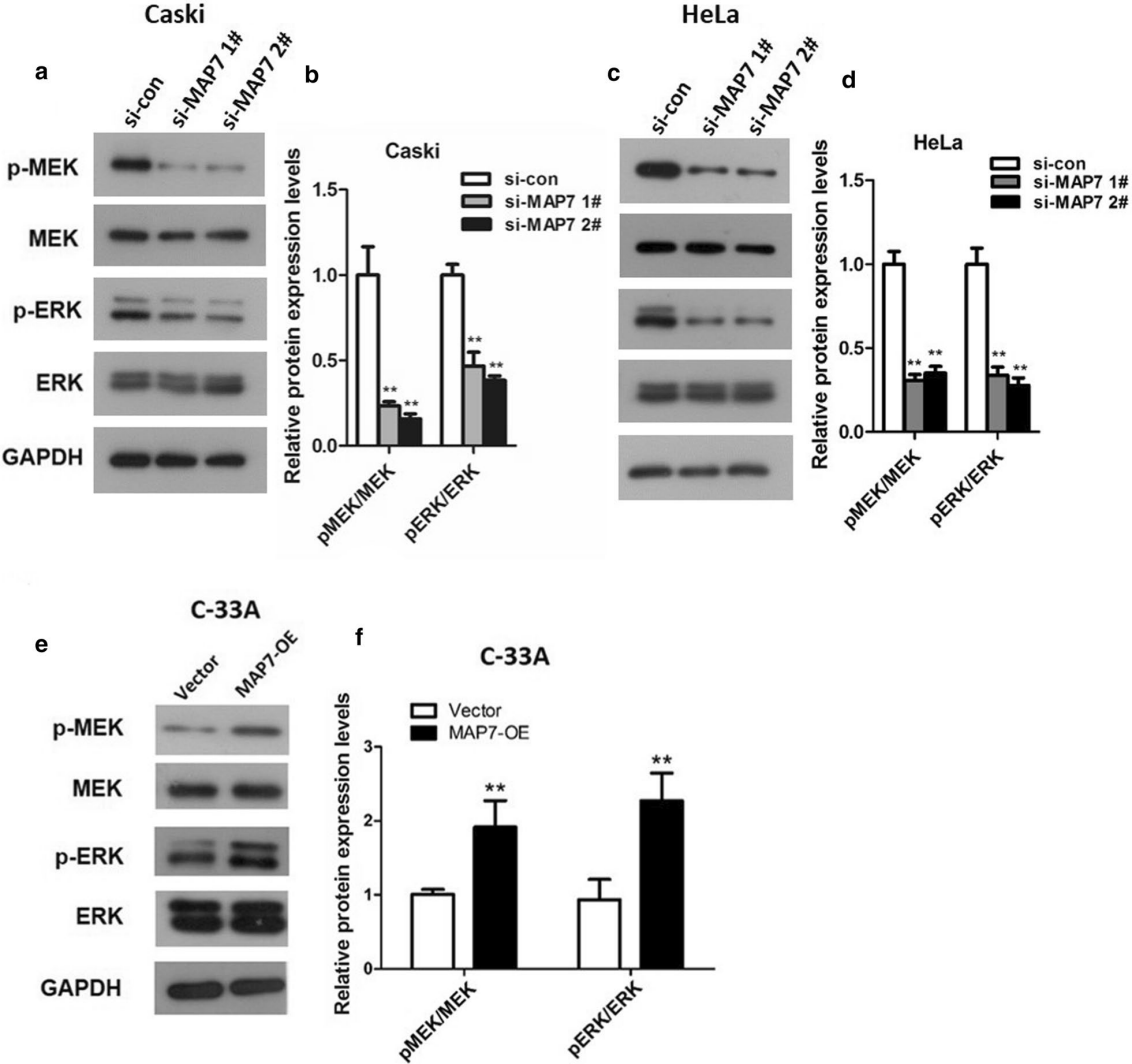
Activation of the MAPK signaling pathway in CC cells transfected with si-MAP7 1#/2# or MAP7-OE. **a**, **c** Western blot analysis of phosphorylated and total MEK and ERK expression in Caski and HeLa cells with MAP7 knockdown; and **b**, **d** the p-MEK/MEK and p-ERK/ERK ratios in Caski and HeLa cells. **e** Western blot analysis of phosphorylated and total MEK and ERK expression in C-33A cells with MAP7 overexpression; and **f** the p-MEK/MEK and p-ERK/ERK ratios in C-33A cells. n = 6; **p < 0.01 vs. controls (si-con or vector). *MAP7* microtubule-associated protein 7, *si-MAP7* siRNA targeting MAP7, *si-con* scrambled siRNA, *MAP7-OE* MAP7-overexpression vector

The MAPK signaling pathway is known to be involved in a number of cellular processes, including proliferation, migration and apoptosis [16–18]. Therefore, activation of this pathway in CC cells was investigated by western blot analysis of phosphorylation of key mediators of this pathway, MEK and ERK (Fig. 8). As presented in Fig. 8a–d, knockdown of MAP7 declined the ratios of p-MEK/MEK and p-ERK/ERK in Caski and HeLa cells (p < 0.01). Overexpression of MAP7 increased the ratios of p-MEK/MEK and p-ERK/ERK in C-33A cells (Fig. 8e–f, p < 0.01). These results suggest that MAP7 expression influences the activation of the MAPK pathway.

### Knockdown of MAP7 inhibited the growth of CC tumors in vivo

**Fig. 9:**
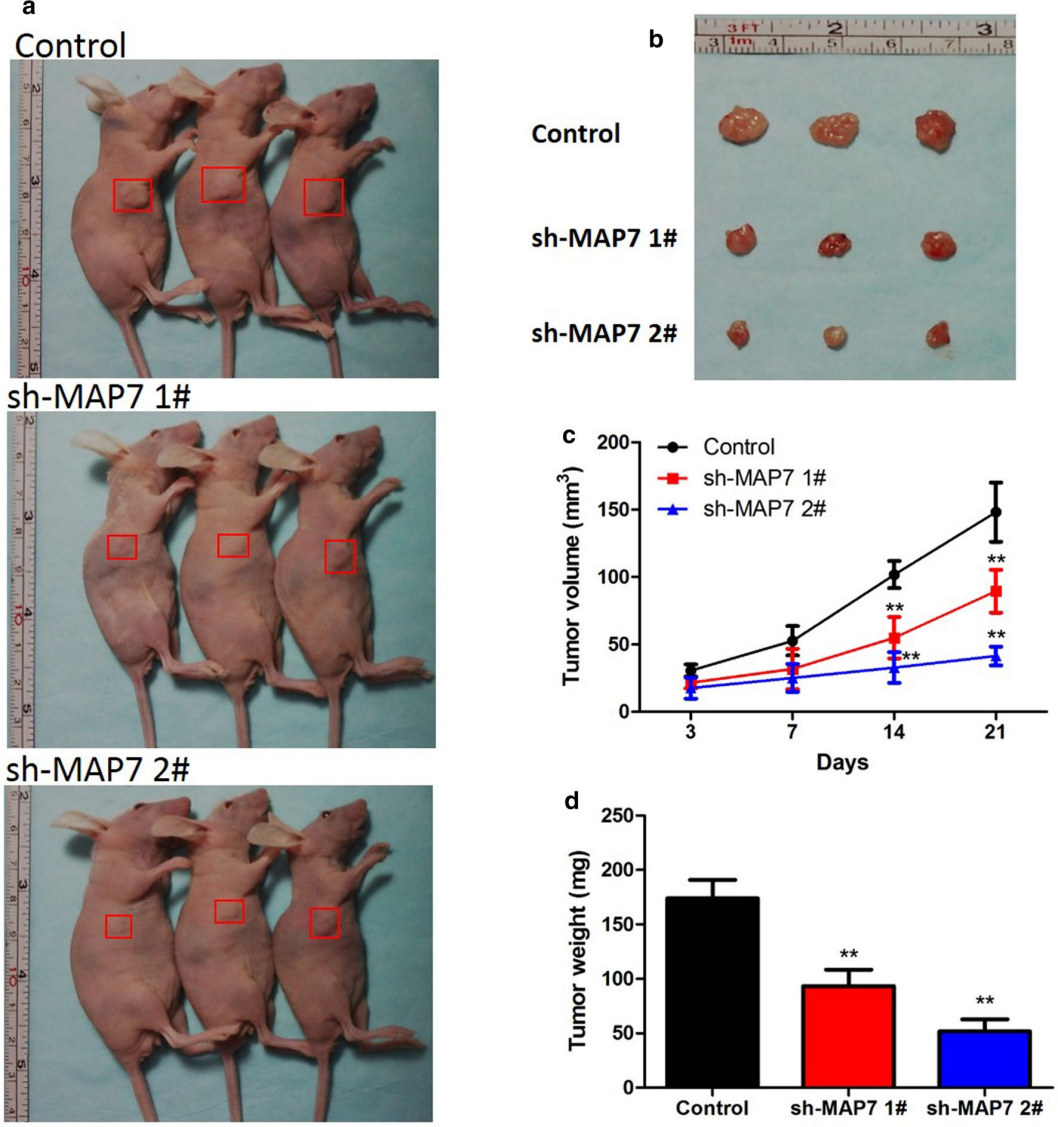
The effect of MAP7 on the growth of CC tumors were detected in mice model in vivo. **a**, **b** The images of mice and the tumors removed from mice after 21 days injection. **c** The growth curves of the tumors. **d** The weight of the tumors. **p < 0.01 vs. Control group

To further prove the promoting effect of MAP7 on CC that we observed in vitro, we next detected the influence of MAP7 on tumor growth in vivo. Caski cells with stable depleted MAP7 and that transfected with negative control were injected into mice. Figure 9a, b presented the photos of the mice and the tumors removed from the mice. The tumor volume was measured at 3, 7, 14, 21 days after injection (Fig. 9c). The weight of tumors were measured after being taken out and were shown in Fig. 9d. Based on the volume and weight of the tumors, we can easily find that knockdown of MAP7 repressed the growth of CC cells in vivo, which was consistent with the results that we obtained in vitro.

## Discussion

In this study, MAP7 was found to be upregulated in CC tissues compared to normal tissues. Further investigations identified that knockdown of MAP7 reduced the viability and motility of Caski and HeLa cells, whilst overexpression of MAP7 in C-33A cells resulted in the opposite effects. Moreover, the results suggest that MAP7 may exert a facilitating role in cell growth and motility by modulating MAPK signaling.

MAP7 is a member of the MAPs family that are associated with microtubule dynamics and known to regulate microtubule functions and involve in epithelial cell differentiation [19]. To date, the function of MAP7 in cancer development has not been fully understood. Herein, the expression of MAP7 was identified to be upregulated in CC tissues, based on publicly-available data. This result was further confirmed by the qRT-PCR performed in CC cell lines and End1/E6E7 cell line, as we observed that the expression of MAP7 was higher in CC cell lines than that in End1/E6E7 cell line. However, due to the lack of normal samples in the public database, there exists a difference between the sample size of tumor group and normal group. More clinical specimens will be collected for validation of the up-regulation of MAP7 in CC in the near future. By Kaplan–Meier analysis, it was demonstrated that high MAP7 level was related with worse prognosis in patients with CC. Moreover, multivariate analysis suggestted that MAP7 expression may serve as an independent predictor for worse survival of patients with CC. Previously, MAP7 was identified one out of 15 genes which were obviously up-regulated in metastatic endometrial cancer using a 22K Affymetrix array [20]. In 2008, CRAIG BLUM et al. have identified that the up-regulation of MAP7 was associated with tumor recurrence and worse prognosis in patients with stage II colon cancer [12]. In young patients with cytogenetically normal acute myeloid leukemia, high MAP7 expression is also predictive of adverse prognosis [11]. These data suggested that MAP7 is a promising predictor for patients with CC and might be act as a promoter in cancer occurrence and progression.

To elucidate the roles and functions of the MAP7 in CC progression, we further explored what effect of MAP7 expression changes may present on CC cell viability, migration, invasion and apoptosis. Our results showed that silencing MAP7 significantly suppressed Caski and HeLa cell viability, colony formation ability, migration and invasion, while enhanced cell apoptosis. On the contrary, overexpression of MAP7 in the C-33A cells presented the opposite outcomes. Moreover, MAP7 declined the levels of pro-apoptotic proteins Active-caspase 3 and Bax whilst increased the level of anti-apoptotic protein Bcl-2. Collectively, MAP7 might play a promoting role in cancer cell growth and motility and exert a repressive role in cell apoptosis, which were consistent with the previous speculation that MAP7 was a promoter in cancer progression. Yan et al. have also found that knockdown of MAP7 markedly decreased A549 cell proliferation, but did not present significant influence in cell apoptosis and cell cycle progression [13]. The different results in cell apoptosis between our and Yan et al’s may be caused by the difference between different tumor cells.

To further explore the possible mechanism of MAP7 regulated cell viability and motility, we studied the effect of knockdown MAP7 on MAPK pathway. We found that the ratios of p-MEK/MEK and p-ERK/ERK decreased in Caski and HeLa cells with down-regulated MAP7 compared to the corresponding controls, whilst the ratios of p-MEK/MEK and p-ERK/ERK raised in C-33A cells with overexpressed MAP7. Unsuitable cell survival and uncontrolled proliferation are among the features of tumor, and these processes are commonly associated with the MAPK/ERK signaling pathway [21–23]. The MAPK cascade is an important intracellular signaling which could response to a serious of stimulation as well as modulate multiple cellular processes including differentiation, proliferation, apoptosis or survival, and inflammation [24]. MAPK (MEK) is a downstream kinase of RAS pathway. When MEK was phosphorylated, it can further activate ERK1/2 [25]. ERK1/2 pathway is one of the most vital and well- characterized sub group of MAPKs family [24]. ERK could facilitate tumor cell migration via phosphorylating calpain, myosin light chain kinase, paxillin, and focal adhesion kinase [26]. ERK signaling also could lead to tumor cell invasion through inducing matrix metalloproteinases expression [27]. In addition, ERK1/2 signaling has been illustrated to modulate the activities and levels of apoptotic-related proteins and then improving the survival of tumor cells [28]. The involvement of MAPK/ERK signaling pathway in CC proliferation and apoptosis has been illustrated by many researchers. ERK1/2 was highly expressed in CC tissues and exhibited a promoting role in CC cell proliferation and a suppressive role cell apoptosis [29]. Our findings indicated that MAP7 promoted the activation of MAPK signaling pathway, implying that MAP7 may promote CC cells viability, motility and repress CC cell apoptosis by activating MAPK signaling pathway. This is the first time that demonstrated the effect of MAP7 expression on MAPK signaling pathway activation. However, how MAP7 talk with MAPK signaling pathway remains to be explored in the future.

Previously research has revealed that MAP7 and its paralogous protein Map7D1 are needed for the adhesion and migration of HeLa cells, and they take part in a feedback loop between microtubule remodeling and Wnt5a pathway via a direct interaction with Disheveled (a vital signal transducer of Wnt signaling) [30]. Moreover, the results obtained by Koji Kikuchi et al. suggested that the role of Map7/7D1 family in the localization of Disheveled is evolutionarily conserved [30]. These data suggested that MAP7 takes part in tumor progression partially via implicating in microtubule remodeling. The molecular mechanisms of tumor progression is quite intricate and one gene often has interactions with various molecules. Hence, lots efforts are still required in the future to achieve a comprehensive understanding on the molecular mechanism of MAP7 in tumor progression.

## Conclusion

In summary, MAP7 was found to be upregulated in CC tissues, and high MAP7 expression was correlated with unfavorable prognosis in patients with CC. Furthermore, the present study demonstrated that MAP7 may act as a promoter of CC progression by regulating the MAPK signaling pathway. These results suggest that MAP7 may serve as a predictor of prognosis, as well as a promising therapeutic target for CC. More detailed mechanism of how MAP7 involved in CC progression deserves our further study.

## Data Availability

The datasets used and/or analyzed during the current study are available from the corresponding author on reasonable request.
